# A Novel *Vpb4* Gene and Its Mutants Exhibiting High Insecticidal Activity Against the *Monolepta hieroglyphica*

**DOI:** 10.3390/toxins17040167

**Published:** 2025-04-01

**Authors:** Ying Zhang, Rongrong Shi, Pengdan Xu, Wei Huang, Chunqin Liu, Jian Wang, Changlong Shu, Jie Zhang, Lili Geng

**Affiliations:** 1 State Key Laboratory for Biology of Plant Diseases and Insect Pests, Institute of Plant Protection, Chinese Academy of Agricultural Sciences, Beijing 100193, China; 2 Hebei Key Laboratory of Soil Entomology, CangZhou Academy of Agriculture and Forestry Sciences, Cangzhou 061001, China; 3 College of Plant Protection, Anhui Agricultural University, Hefei 230036, China; 4 China National Seed Group Co., Ltd., Sanya 572000, China; 5 National Center of Technology Innovation for Maize, Beijing 100101, China

**Keywords:** *Bacillus thuringiensis*, *Monolepta hieroglyphica*, vpb4Fa1, random mutation

## Abstract

*Monolepta hieroglyphica* Motschulsky, a major agricultural pest in China, causes considerable economic damage to crops, such as maize. In this study, a *Bacillus thuringiensis* (Bt) strain was discovered to exhibit insecticidal activity against *M. hieroglyphica*. A novel Bt gene, *vpb4Fa1*, with toxicity against both adults and larvae of *M. hieroglyphica* was cloned. The Vpb4Fa1 protein causes damage to the midgut of adult *M. hieroglyphica*, disrupting their normal growth and development and ultimately leading to death. To further enhance the insecticidal activity of the *vpb4Fa1* gene, a random mutation library was established. A total of 75 mutants with amino acid mutations were generated, among which 7 mutants demonstrated significantly enhanced activity relative to the wild-type gene. Notably, three mutants, C9, 6C2, and 6A7, exhibited the highest activity, with LC_50_ values for adult *M. hieroglyphica* of 10.21, 9.45, and 9.83 µg/g, respectively. The mutants C9, 6C2, and 6A7 each harbored nine, three, and six amino acid mutations, respectively, mainly located in Domains I, II, and III. The novel insecticidal gene *vpb4Fa1* and its mutants offer valuable genetic resources for the biological control of *M. hieroglyphica* and the development of Bt transgenic maize.

## 1. Introduction

Corn is the top staple crop in China, with an annual production of approximately 500 million tons, accounting for about 10% of the country’s total grain output. It plays a strategic role in ensuring national food security [1]. In recent years, the spread and extent of damage caused by *Monolepta hieroglyphica* Motschulsky have been increasing, making it one of the key pests of crops such as corn in various regions of China [2]. During peak infestation periods, the damage rate can reach up to 100%, resulting in a severe decline in corn yields, with complete crop failure occurring under severe infestation conditions [3,4,5,6]. *M. hieroglyphica* belongs to the Galerucinae subfamily of the Chrysomelidae family within the Coleoptera order. This pest is widely distributed across China, and is a polyphagous insect with a broad host range, primarily feeding on plants from the Poaceae, Brassicaceae, and Fabaceae families [7,8]. Key characteristics of this pest include its gregarious behavior, extended damage period, rapid reproduction, and short-distance migration. Both the larvae and adults of *M. hieroglyphica* cause significant damage to corn. The larvae live in the soil, feeding on the corn roots, while the adults exhibit a gregarious feeding behavior [9].

Currently, chemical pesticides are the primary method for controlling *M. hieroglyphica*, with commonly employed agents including chlorpyrifos, thiamethoxam, deltamethrin, and fipronil [2,10]. However, the long-term overuse of chemical pesticides poses risks such as environmental pollution and the development of pesticide resistance in target pests. Presently, there have been few reports on the biological control of *M. hieroglyphica*. Effective biopesticides include abamectin and the entomopathogenic fungus *Metarhizium anisopliae* strain CQMa421 [11,12]. Natural enemies of *M. hieroglyphica* include ladybugs, spiders, and parasitic wasps, but they have not been widely applied in field control [13]. Due to the lack of a well-established indoor rearing system and bioassay protocols for *M. hieroglyphica*, no effective insecticidal genes have been reported so far.

*Bacillus thuringiensis* (Bt) is a Gram-positive bacterium belonging to the Bacillaceae family. It is commonly found in natural environments such as soil, water, insects, and plant surfaces [14,15]. During its growth, Bt produces various insecticidal proteins including insecticidal crystal proteins (ICPs) such as Cry and Cyt proteins, which are formed during the late stage of sporulation [16]; vegetative insecticidal proteins (Vip) produced during the vegetative growth phase [17]; and secreted proteins (Sip) [18]. Bt exhibits insecticidal activity against a wide range of insects, including Lepidoptera, Coleoptera, Hymenoptera, and Diptera, as well as against mites and nematodes [19,20]. Currently, Cry3 [21], Cry5, Cry7, Cry8 [22], Cry18, Vip1/Vip2 [23], and Vpb4 have been reported to exhibit insecticidal activity against Coleopteran pests.

The *vpb4* gene family consists six genes, including four model genes: *vpb4Aa1*, *vpb4Ba1*, *vpb4Ca1*, and *vpb4Da1*. There are limited reports on the Vpb4 protein, among which the Vpb4Da2 protein contains four structural domains: the PA14 domain, and the Binary_toxB, toxB_2, and toxB_3 domains. Vpb4Da2 exhibits specific insecticidal activity against the western corn rootworm (WCR), *Diabrotica virgifera virgifera* (LeConte), which also belongs to the Galerucinae subfamily of the Chrysomelidae family within the order of Coleoptera. At a concentration of 31.25 µg/cm^2^, Vpb4Da2 showed strong growth inhibition and significant insecticidal activity against WCR larvae. The cultivation of Vpb4Da2 transgenic corn has been shown to reduce root damage caused by WCR larvae and lowers both the survival rate and emergence rate of WCR [24]. Feeding WCR with the Vpb4Da2 protein caused structural damage to the midgut epithelial cells, including the rupture of the apical microvilli, with detachment of lumen cellular debris [25].

Error-prone PCR is one of the most commonly used method for inducing random mutations. This method primarily relies on the use of Taq DNA polymerase during PCR amplification. By adjusting reaction conditions, such as modifying the magnesium ion concentration, incorporating manganese ions, and altering the number of PCR cycles, the probability of incorrect base incorporation is increased, thereby inducing random mutations in the target gene. Shu et al. used error-prone PCR to create a random mutation library of Cry8Ca, resulting in two mutants, M100 (E642G) and M102 (Q439P, E884G), which showed 4-fold and 4.5-fold increases in toxicity against *Anomala corpulenta* Motschulsky (Coleoptera: Scarabaeidae), respectively [26]. Kumar et al. constructed a random mutation library of Cry1Ac and identified mutants with a 3-fold increase in toxicity against *Manduca sexta* Joh. (Lepidoptera: Sphingidae) and *Helicoverpa assulta* Guenée (Lepidoptera: Noctuidae) [27].

In this study, a wild-type Bt strain, B14D2, with insecticidal activity against both adults and larvae of *M. hieroglyphica*, was obtained from a Bt library stored in our lab. The novel *vpb4Fa1* gene was cloned, and the protein caused damage to the midgut of adult *M. hieroglyphica*. A random mutation library of Vpb4Fa1 was successfully constructed, and seven mutants with significantly enhanced insecticidal activity against *M. hieroglyphica* were selected, providing a new strategy for the control of this pest.

## 2. Results

### 2.1. Insecticidal Activity of Bt B14D2 Strain Against M. hieroglyphica

The Bt B14D2 cells were collected, and a bioassay was performed at a concentration of 10^8^ cfu/mL on *M. hieroglyphica*. The corrected mortality rate for adult *M. hieroglyphica* was 22.2%, while the larvae exhibited insecticidal activity with a corrected mortality rate of 40.4%. Further analysis of the insecticidal spectrum revealed that the B14D2 strain showed high toxicity against *Mythimna separata* Walker (Lepidoptera: Noctuidae), *Helicoverpa armigera* Hübner (Lepidoptera: Noctuidae), and *Plutella xylostella* Linnaeus (Lepidoptera: Plutellidae) (Table 1).

### 2.2. Whole Genome Sequencing Analysis of Bt B14D2 Strain

**Figure 1:**
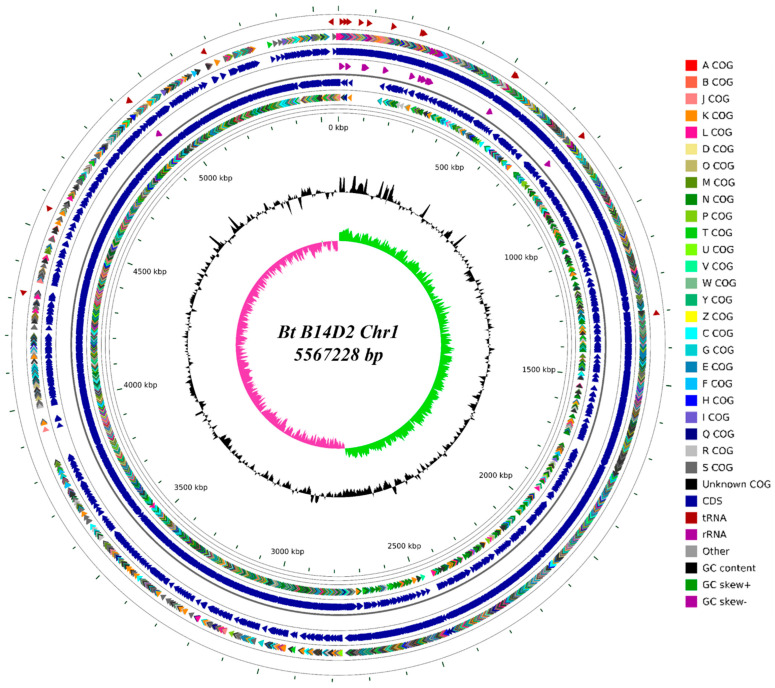
Circos plot of B14D2. The layers, progressing from the outermost to the innermost, are as follows: tRNA-related genes; COG annotations of forward-strand genes, distinguished by different colors (refer to the right-side description for details); positions of forward-strand genes; rRNA genes; coordinates of reverse-strand genes; COG annotations of reverse-strand genes; GC content, with the average GC content as the baseline—protruding outward indicates values above the average, while protruding inward indicates values below the average; the innermost circle represents the GC skew values, where purple indicates values less than 0 and green indicates values greater than 0.

**Figure 2:**
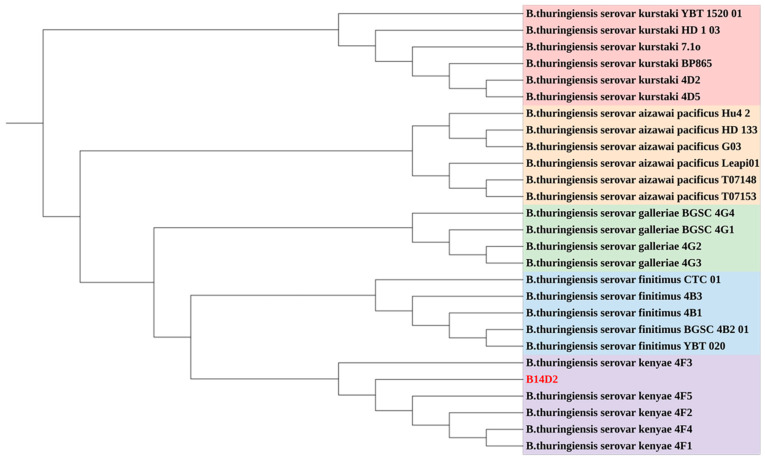
CVTree phylogenetic analysis of Bt B14D2 strain.

Whole genome sequencing of the Bt B14D2 strain was performed (Figure 1). The assembly resulted in two circular contigs and four linear contigs, consisting of one circular chromosome and five plasmids. The genome size is 5.48 Mb, with a total of 6,405,341 base pairs and a GC content of 34.99%. The Bt B14D2 strain contains 6789 coding genes, of which 6487 are protein-coding sequences (CDS). Phylogenetic analysis using the genome data of Bt B14D2 and 37 reference strains was performed with CVTree (Figure 2). The results indicated that Bt B14D2 belongs to the Bt subsp. *kenyae*.

Further annotation of the whole genome sequence of Bt B14D2 was performed for insecticidal genes (Table 2), identifying four insecticidal genes. Sequence alignment revealed that Bt B14D2 contains *cry1Ea3*, *cry2Ab4*, *vip3Aa59*, and a novel *vpb4* gene. The protein encoded by the *vpb4* gene shares 53.50% amino acid similarity with Vpb4Da2, which has been reported to exhibit specific insecticidal activity against WCR larvae [24].

### 2.3. Expression and Bioassay Analysis of Vpb4Fa1 Protein

**Figure 3:**
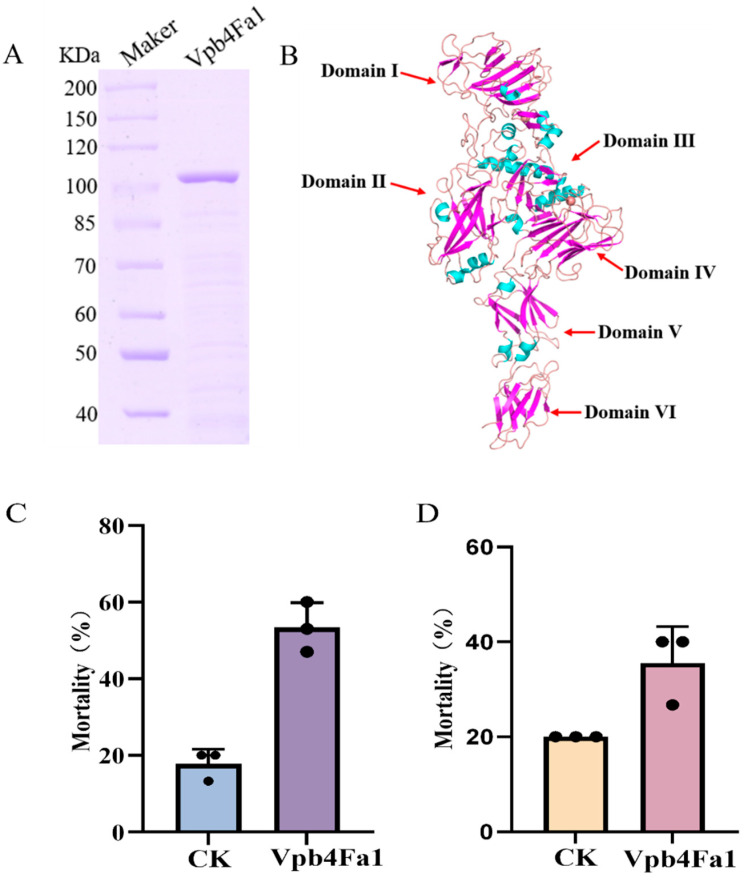
Purification and bioassay of Vpb4Fa1 protein. (**A**) Purification of Vpb4Fa1 protein. Maker: 26614; (**B**) 3D structure prediction of Vpb4Fa1 protein (SWISS-MODEL), blue represents α-helix, light pink represents loops, and dark pink represents β-sheet. (**C**) Bioassay of Vpb4Fa1 protein against *M. hieroglyphica* adults. (**D**) Bioassay of Vpb4Fa1 protein against *M. hieroglyphica* larvae. CK: *M. hieroglyphica* adults were fed with 20 mM Tris-HCL; the concentration of Vpb4Fa1 protein is 40 μg/g. The original data were presented in Table S4.

A novel *vpb4* gene was cloned, and a 114 kDa protein was successfully expressed in *Escherichia coli* BL21. The protein was subsequently desalted and purified (Figure 3A). According to the SDS-PAGE results, the purity of Vpb4Fa1 protein is 92.8%. The gene was submitted to BPPRC (https://www.bpprc-db.org/view_cart/, accessed on 23 October 2024), and according to the system for naming Bt proteins, it was designated as Vpb4Fa1 (GenBank No: PQ478525). Analysis of the amino acid sequence of the Vpb4Fa1 protein revealed that it contains the following domains, PA14, Binary_toxB, toxB_2, and toxB_3, located at amino acid residues 45–170, 210–286, 288–502, and 508–609, respectively. These domains are consistent with those found in Vpb4Da2, a protein previously reported to exhibit insecticidal activity against WCR. The Vpb4Fa1 protein model was constructed using the SWISS-MODEL online software (https://swissmodel.expasy.org/, accessed on 10 October 2024, Figure 3B), with reference to the Vpb4Da2 domain structure [25]. Based on this, the Vpb4Fa1 protein can be roughly divided into six domains: Domain I (amino acids 1–299), Domain II (amino acids 300–517), Domain III (amino acids 518–627), Domain IV (amino acids 628–764), Domain V (amino acids 765–854), and Domain VI (amino acids 855–950). The bioassay results showed that at a concentration of 40 µg/g, the Vpb4Fa1 protein exhibited a corrected mortality rate of 43.5% against *M. hieroglyphica* adults (Figure 3C) and a corrected mortality rate of 19.2% against larvae (Figure 3D).

**Figure 4:**
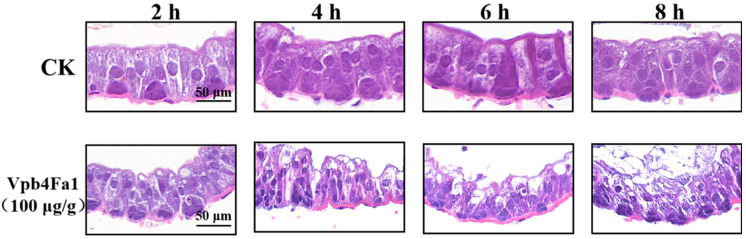
A temporal sequence of histopathological effects induced by Vpb4Fa1 protein on the midgut of *M. hieroglyphica*. CK: *M. hieroglyphica* adults were fed with 20 mM Tris-HCL. The microscope models are Pannoramic MIDI (3DHISTECH, Budapest, Hungary), Pannoramic 250FLASH, and Pannoramic DESK (Observations were made using a 20× objective lens).

After feeding *M. hieroglyphica* adults with Vpb4Fa1 protein at a concentration of 100 µg/g, the midgut of the adults was dissected at different time points. Significant damage to the midgut was observed (Figure 4). In the control group, the midgut epithelial cells of *M. hieroglyphica*, which were not fed with Bt protein, appeared oval-shaped and were arranged in a dense, orderly pattern. After 2 h of Vpb4Fa1 protein treatment, the cells elongated, began to deform, and were arranged in a disordered pattern. After 4–6 h, tissue damage intensified, with cells becoming more loosely arranged and disorganized, and vacuolation began to appear. After 8 h, cell morphology became difficult to distinguish, with extensive vacuolation and rupture of the epithelial cells. After 10 h, part of the apical microvilli layer was disrupted.

### 2.4. Establishment of the Vpb4Fa1 Random Mutant Library

Excessive numbers of mutated bases can result in a high frequency of nonsense mutations. To increase the proportion of sense mutations, we set the number of mutated bases to two. Using a formula, we determined that 26 PCR cycles were required to induce mutations in two bases. The initial template amount for constructing the mutant library was set at 4 ng, as determined by gradient PCR. A random Vpb4Fa1 mutant library containing 103 single clones was constructed, yielding 102 mutants with altered nucleotide sequences, resulting in a base mutation rate of 99.1%. Statistical analysis of the mutation sites in the Vpb4Fa1 random mutant library showed a relatively uniform distribution. A total of three mutation hotspots were identified: base 666 changed from A to G in 75 mutants, base 931 changed from T to C in 73 mutants, and base 2292 changed from T to C in 73 mutants. Amino acid sequence analysis revealed a mutation rate of 99.1%, with nonsense mutations accounting for 30.3% (33 mutants in total), missense mutations accounting for 68.6% (75 mutants in total), and one mutant remaining unchanged.

**Figure 5:**
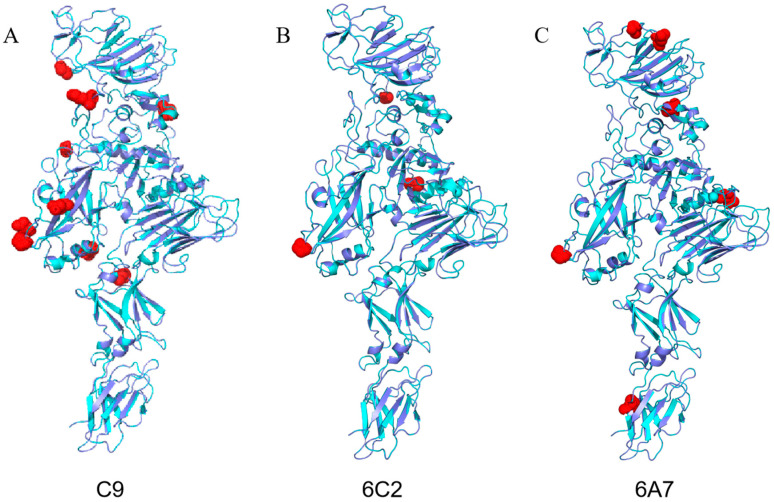
PyMOL 3D structure prediction of Vpb4Fa1 mutant proteins. (**A**) C9. (**B**) 6C2. (**C**) 6A7. Dark blue: 3D structure of Vpb4Fa1; light blue: 3D structure of mutant proteins; red: mutation sites.

The mutants were introduced into *E. coli* BL21 competent cells for protein expression. SDS-PAGE results showed that 75 mutants successfully expressed a 114 kDa protein (Figure S1). When the mutant proteins were fed to *M. hieroglyphica* adults at a concentration of 10 µg/g, initial screening revealed seven mutants with significantly increased insecticidal activity (Table S5). The seven mutants were further analyzed, and the corrected mortality rate for adults ranged from 60.7% to 82.1% (Table 3). Based on the bioassay results, three mutants were selected for further testing on *M. hieroglyphica* adults to determine the lethal concentration (LC_50_). Specifically, mutant C9 was tested at concentrations of 1, 2, 4, 8, 16, and 32 µg/g, while mutants 6A7 and 6C2 were evaluated at concentrations of 1.75, 2.25, 4.5, 9, 18, and 36 µg/g. As in the previous experiments, 20 mM Tris-HCl was used as the control. The LC_50_ for mutant C9 was 10.21 µg/g (95% confidence interval: 5.06–21.26 µg/g), for mutant 6C2 was 9.45 µg/g (95% confidence interval: 7.86–11.35 µg/g), and for mutant 6A7 was 9.83 µg/g (95% confidence interval: 7.14–13.52 µg/g) (Table S6). The toxicity of these three mutants against *M. hieroglyphica* larvae was also analyzed. At a protein concentration of 10 µg/g, the corrected mortality rates for mutants C9, 6C2, and 6A7 were 41.7%, 50%, and 36.1% (Table S7), respectively. The three-dimensional structural models of these three proteins were constructed using PyMOL. The mutation sites were primarily located in Domains I, II, and III (Figure 5). These domains play key roles in the protein’s function and stability. Therefore, the distribution of mutation sites may significantly impact on the protein’s activity and conformational stability, affecting its interactions with target molecules and consequently altering its insecticidal activity.

## 3. Discussion

The damage caused by *M. hieroglyphica* on maize has been increasingly severe, gradually becoming an important emerging pest in maize production in regions such as southern Shandong and Shanxi. In this study, the strain B14D2 exhibits insecticidal activity against both adult and larval *M. hieroglyphica*, a discovery that not only enriches the Bt strain library, but also provides new resources for biological control. Furthermore, this study demonstrates the efficacy of the strain B14D2 against *P. xylostella*, *H. armigera*, and *M. separata*. Currently, most *Bacillus thuringiensis* (Bt) strains demonstrate insecticidal activity against a single target pest, either Coleopteran or Lepidopteran. To broaden the insecticidal spectrum, recombinant Bt strains have been developed, such as G033A, which exhibits dual insecticidal activity against both Lepidopteran and Coleopteran pests [28]. Furthermore, the total protein extract derived from the wild-type strain B14D2 has shown insecticidal activity against both the Coleopteran pest *M. hieroglyphica* and the Lepidopteran pest *P. xylostella*. These findings offer valuable strain resources for overcoming the limitations of Bt’s insecticidal spectrum and hold significant promise for biological control and integrated pest management (IPM).

According to the latest nomenclature, Vpb proteins are classified into four groups based on amino acid homology, with similarity thresholds set at 45%, 78%, and 95% [29]. Currently, the *vpb4* gene family consists of six genes, including four model genes: *vpb4Aa1*, *vpb4Ba1*, *vpb4Ca1*, and *vpb4Da1*. The novel *vpb4* gene cloned in this study encodes a protein with 53.5% amino acid similarity to the Vpb4Da protein. This gene has been submitted to BPPRC and designated as a new model gene, *vpb4Fa1*. Additionally, the identification of the novel *vpb4Fa1* gene and mutations showed high toxicity, offering a new research direction for controlling *M. hieroglyphica*. Numerous studies have demonstrated that insecticidal proteins expressed by Bt strains do not negatively impact non-target insects, such as pollinators, natural enemies, and soil-dwelling insects. For example, the Bt insecticidal genes *cry1Ab* and *cry1Ac*, which are widely used in transgenic insect-resistant crops, have been shown to have no adverse effects on the growth and development of pollinators [30,31]. However, whether the Vpb4Fa1 protein affects non-target insects remains uncertain, highlighting the need for further laboratory validation. The discovery of this novel gene provides a valuable genetic resource for controlling *M. hieroglyphica* and offers new research avenues for managing *D. virgifera virgifera* while also helping to delay the development of pest resistance. The toxicity of Vpb4Fa1 against *D. virgifera virgifera* needs further analysis.

After being ingested by insects, Bt insecticidal proteins are activated in their bodies and bind to target sites on the midgut epithelial cells. This binding causes cell perforation, typically leading to pathological ultrastructural changes in the insect’s midgut. These changes are characterized by cell deformation, the widening of intercellular spaces, and the swelling and detachment of microvilli. [32,33]. After feeding on Vpb4Fa1 protein, *M. hieroglyphica* adults exhibited noticeable deformation of midgut epithelial cells. Vacuolization was observed between the cells, and in cases of severe damage, the cell boundaries became unclear, with midgut epithelial cells detaching into the gut lumen. In the Coleoptera order, Cry3A protein feeding by *Zygogramma bicolorata* Pallister [34], Bt strains HD8E and HD8G feeding by *Phyllophaga crinite* Burmeister [35], and Bt strain HBF-1 feeding by *Anomala cuprea* Hope all resulted in midgut pathological changes, consistent with those observed in this study [36].

Bt insecticidal proteins can be modified through random mutagenesis, and high-throughput sequencing combined with bioassays can be used to screen and identify mutants with enhanced activity. This study performed random mutagenesis on the *vpb4Fa1* gene to obtain mutants with a low number of base mutations and enhanced toxicity. Sequencing results revealed a total of 75 mutants with amino acid sequence changes. Corresponding proteins were extracted, and bioassays on *M. hieroglyphica* adults identified seven mutants with significantly enhanced activity. The high-activity mutants showed mutations mainly located in Domains I, II, and III, while mutants with reduced activity had mutations in Domains I, II, and VI. According to Kouadio’s study, Domains I and III of Vpb4Da2 protein are involved in protein oligomerization, while Domain II acts as a potential pore-forming region, playing a crucial role in membrane pore formation. Domains IV, V, and VI play a crucial role play a crucial role in insecticidal activity. The deletion of these three domains reduces the insecticidal activity of Vpb4Da2 [25]. Based on the structural and functional similarities between Vpb4Fa1 and Vpb4Da2, mutations in Domains I and III likely enhance insecticidal activity by improving the protein’s oligomerization properties. Meanwhile, Domain II, as a potential pore-forming region, may alter the protein’s interaction with membranes, thereby enhancing its pore-forming ability. This ultimately results in the leakage of cellular contents and insect mortality. Mutations in Domain VI directly lead to the loss of insecticidal activity. While the current study sheds light on the potential mechanisms of these proteins, the specific molecular mechanisms still require further exploration.

This study first identifies the novel Vpb4Fa1 protein as having high insecticidal activity against *M. hieroglyphica*, capable of inducing pathological changes in the insect’s midgut. To further enhance its toxicity, we applied random mutagenesis to modify the *vpb4Fa1* gene and successfully obtained mutants with significantly improved insecticidal activity. However, several issues remain to be addressed: (1) The binding receptor of Vpb4Fa1 protein with the *M. hieroglyphica* is still unclear; (2) The molecular mechanisms linking the mutated sites in the protein to the enhanced insecticidal activity require further investigation. While Vpb4Fa1 demonstrates significant insecticidal potential, it also provides a new genetic resource for the development of Bt transgenic plants. (3) The activity spectrum and potential on non-target organisms are still uncertain. Overall, Vpb4Fa1 not only shows great insecticidal promise, but also offers new avenues for the development of Bt-based transgenic crops.

## 4. Conclusions

In this study, a Bt strain, B14D2, was found to exhibit insecticidal activity against both adult and larvae of *M. hieroglyphica*. High-throughput sequencing identified a novel *vpb4Fa1* gene, which demonstrated high insecticidal activity against *M. hieroglyphica*. This gene causes damage to the midgut of adult *M. hieroglyphica*. To enhance the insecticidal activity of the *vpb4Fa1* gene, a random mutagenesis library was constructed, and seven mutants with improved activity were obtained. These findings contribute to expanding the genetic resources of Bt insecticidal proteins and provide new approaches for developing novel biopesticides. Furthermore, they offer new research directions and technological solutions for controlling *M. hieroglyphica*.

## 5. Materials and Methods

### 5.1. Bacterial Strains and Culture Conditions

The bacterial strain B14D2 was cultured on 1/2 LB solid medium at 30 °C for 48 h, followed by plate counting. The bacterial cells were collected when the concentration reached 10^8^ cfu/mL. *E. coli* strains were cultured in LB liquid medium at 37 °C with shaking at 220 rpm for 12 h.

### 5.2. Genomic DNA Extraction and Sequencing

Genomic DNA was extracted using the CTAB method [37]. The whole genome was sequenced by Sinobiocore (Beijing, China) using the PacBio sequencing platform. Assembly was carried out with HGAP [38]. CDS prediction was carried out using Prodigal [39]. A GC plot was generated based on the genome sequence. The distribution of COG-annotated genes on the genome was mapped according to the COG annotation results [40] and the positional information of the genes.

The Bt insecticidal protein database, which includes publicly available Bt insecticidal protein sequences from NCBI, was established using BioEdit (v7.2.6.1). The predicted amino acid sequences of the strains were input into BLAST for local alignment, with an E-value cutoff set at 10^−5^, to annotate the strain’s amino acid sequences.

### 5.3. Establishment of the Random Mutation Library for the vpb4Fa1 Gene and Protein Extraction

The bacterial strain was inoculated into an LB liquid medium and cultured at 30 °C, 220 rpm for 8–12 h. Plasmid extraction was performed using the Plasmid Mini Kit Ι (OMEGA, Guangzhou, China). The plasmid concentration was adjusted to 4 ng/µL, and 1 µL of plasmid was used as the PCR template. Primers were designed based on the *vpb4Fa1* gene sequence, forward primer: TGGTGGTGGTGGTGGTGCTCGAGAATGAAATTAAAAA GTATATTTA, reverse primer: GCGGATATTTTTATCCATTTAAAAGTCGACGGAGCT CGAATTCGGATCCGC. PCR amplification was performed using 2× Taq PCR MasterMix (Biomed, Beijing, China). The PCR cycling conditions were as follows: initial denaturation at 94 °C for 10 min, denaturation at 94 °C for 30 s, annealing at 55 °C for 30 s, extension at 72 °C for 3 min and 10 s, 26 cycles, and final extension at 72 °C for 5 min. The amplified *vpb4Fa1* gene was ligated into the pET-28a vector and sent to Beijing Qingke Technology Co., Ltd. (Beijing, China) and Genechem Biotech (Shanghai, China) Co., Ltd. for sequencing. After sequencing was completed, the mutant strains with the expected mutations were transformed into *E. coli* BL21 (DE3) competent cells.

When the bacterial culture reached an OD_600_ of 0.6, 0.5 mM isopropyl-β-D-thiogalactopyranoside (IPTG) was added to induce expression at 16 °C for 36 h. The culture was centrifuged at 8000× *g* for 10 min to obtain the pellet, which was resuspended in 20 mM Tris-HCl buffer. The suspension was sonicated, and the supernatant was collected. After purification using a Ni-affinity column, desalting was performed using an AKTA system to obtain pure Vpb4Fa1 protein. The protein was then analyzed by 10% SDS-PAGE.

### 5.4. Histopathological Changes in the Midgut of M. hieroglyphica

The adults of *M. hieroglyphica* were fed with 100 µg/g of Vpb4Fa1 protein, with adults fed with a control diet containing 20 mM Tris-HCl serving as the negative control. The adult *M. hieroglyphica* were reared in an artificial climate chamber with a temperature of (25 ± 1) °C, 60% relative humidity, and a light/dark cycle of 16:8. After 2, 4, 6, and 8 h of feeding, dissections were performed to isolate the midgut. The samples were stored in 4% tissue fixative and sent to Jiaves (Wuhan) Biopharmaceutical Co., Ltd. (Wuhan, China) for paraffin embedding and hematoxylin and eosin (H&E) staining. Midgut tissues differences were examined using a panoramic slice scanner (Pannoramic DESK/MIDI/250).

### 5.5. Bioassay Analysis

Strain B14D2 was cultured on 1/2 LB at 30 °C until more than 50% of the bacterial cell lysis was completed. The bacterial cells were scraped into a sterile 50 mL centrifuge tube, thoroughly washed with sterile water, and 4 mL of pre-cooled 50 mmol/L Na_2_CO_3_ (pH 10.0) was added. For the bioassay of *M. separata*, the protein concentrations of 0.0625, 0.125, 0.25, 0.5, and 1 µg/g were used. For the bioassay of *H. armigera* and *P. xylostella*, protein concentrations of 10 µg/g were used. The control group was fed with 50 mmol/L Na_2_CO_3_ (pH 10.0). The aforementioned protein concentrations were determined based on a 130 kDa protein. For the bioassay of *M. hieroglyphica*, a concentration of 10^8^ cfu/mL of the B14D2 strain was used, and the control group was fed with sterile water. The Vpb4Fa1 and mutant proteins were purified for the bioassay of *M. hieroglyphica* and the control group was fed with 20 mM Tris-HCL.

The bioassay for *P. xylostella* was conducted following the leaf-dipping method described by Gao [41]. *P. xylostella* was initially collected from Langfang, China, and has been continuously reared in our laboratory. Cabbage leaves were dipped in a sample suspension containing 0.1% surfactant (Solabio, Beijing, China) for 5 min and air-dried in a Petri dish. Each cabbage leaf was inoculated with 30 first-instar larvae, with each treatment repeated three times. Mortality was recorded after 48 h. The Petri dishes were placed in an artificial climate chamber set at 26 °C, 50% relative humidity, and a light/dark cycle of 16:8.

*M. separata* and *H. armigera* were initially collected from Jilin, China, and were reared by the Jilin Academy of Agricultural Sciences. The bioassay method for *M. separata* and *H. armigera* was adapted from Xue et al. [42]. Artificial diets containing soy flour, wheat bran, yeast, and vitamins were used [43]. After the moisture evaporated at room temperature for 30 min, the diet was evenly distributed into a 24-well plate with a width of 8 cm, a length of 12 cm, and each well having a diameter of 2 cm. The 24 first-instar larvae were introduced into each well and covered with paper towels and lids. Each treatment was replicated three times, with 24 larvae per replicate. The larvae and diets were observed daily, and the corrected mortality rate was calculated after 7 days. The Petri dishes were placed in an artificial climate chamber set at 26 °C, 50% relative humidity, and a light/dark cycle of 16:8.

*M. hieroglyphica* were initially collected from Shanxi, China, and reared by the Plant Protection Research Institute of the Cangzhou Academy of Agricultural and Forestry Sciences. The artificial diet for adult *M. hieroglyphica* contains 5.54% corn flour, 4.77% yeast powder, 1.26% sucrose, 0.30% ascorbic acid, 0.15% ethyl paraben, 0.15% sorbic acid, and 1% agar, which is then mixed and dissolved in 1 L of water, while the larval diet was a mixture of the WCR larval diet WCRMO-2 and F9800B (Frontier Agricultural Sciences, Newark, NJ, USA). The artificial diet was placed in each Petri dish, and the test solution was mixed with the diet. After the moisture evaporated, the diet was evenly distributed. Transparent plastic jars, with a height of 10 cm and a diameter of 8.5 cm, were used for rearing adult insects, while Petri dishes with a diameter of 6 cm were used for rearing larvae. *M. hieroglyphica* of similar size and good health were selected. Each treatment was replicated three times, with 15 adults or first-instar larvae per replicate. The transparent plastic jars or Petri dishes were placed in an artificial climate chamber set at (25 ± 1) °C, with 60% relative humidity and a light/dark cycle of 16:8. The insects and diets were monitored daily, and the corrected mortality rate was calculated after 7 days.

The LC_50_ value was calculated using SPSS (version 19.0) based on Finney’s probit analysis method [44]. Other statistical analyses were performed using GraphPad Prism 9.0 software. Corrected mortality = [(treatment mortality − control mortality)/(100 − control mortality)] × 100. Detailed bioassay data are provided in the Supplementary Tables.

## Figures and Tables

**Table 1:** Insecticidal activity of Bt B14D2 strain against different pests.

Insect	Order	Corrected Mortality (Mean ± SD, %)	LC_50_ (μg/g)	95% Confidence Interval (μg)
*M. hieroglyphica*	Coleoptera	22.2 ± 3.87 (Adults) *	/	/
		40.4 ± 12.01 (Larvae) *	/	/
*M. separata*	Lepidoptera	/	0.29	0.127~0.706
*H. armigera*	Lepidoptera	93.0 ± 2.32 **		/
*P. xylostella*	Lepidoptera	98.8 ± 2.01 **		/

* The concentration of Bt B14D2 strain at 10^8^ cfu/mL. ** The protein concentration is 10 µg/g. The original data were presented in Tables S1–S3.

**Table 2:** Insecticidal gene annotation of Bt B14D2 strain.

Gene	Base	Amino Acids	Station	Alignment
*cry1Ea*	3516	1171	Plasmid4	Cry1Ea1 (100%)
*cry2Ab*	1902	633	Plasmid2	Cry2Ab4 (100%)
*vip3Aa*	2370	789	Plasmid2	Vip3Aa59 (100%)
*vpb4Da*	2931	976	Plasmid1	VpbDa2 (53.5%)

**Table 3:** Bioassay of adult *M. hieroglyphica* (partial).

Treatment	Corrected Mortality (Mean ± SD, %)	Mutation Site	Mutated Amino Acids	Domain
4C10	69.44 ± 4.81	311	S-P	II
		905	L-P	VI
		963	S-P	
6C2	62.07 ± 15.8	182	E-G	Ι
		311	S-P	II
		617	I-V	III
6A7	65.52 ± 11.95	110	N-D	Ι
		126	M-V	
		233	I-T	
		311	S-P	II
		604	K-E	III
		878	V-I	VI
4G7	60.71 ± 6.19	311	S-P	II
		397	P-S	
		440	N-S	
6D7	64.29 ± 16.37	311	S-P	II
		353	G-R	
		892	F-S	VI
4C6	60.71 ± 12.37	205	I-V	Ι
		311	S-P	II
C9	82.14 ± 12.37	41	K-E	Ι
		135	E-G	
		225	G-D	
		305	N-D	II
		311	S-P	
		387	N-D	
		400	D-G	
		466	A-V	
		803	V-L	V
4H12	0 ± 0	218	P-L	Ι
		311	S-P	II
		593	E-G	III
		735	K-E	IV
		887	H-R	VI
5A8	0 ± 0	71	L-P	Ι
		311	S-P	II
		930	F-S	VI
H4	2.78 ± 4.81	84	I-V	Ι
		311	S-P	II
2C2	5.56 ± 9.62	288	Y-C	Ι
		311	S-P	II
		674	Y-H	VI
WT	16.67 ± 8.33	/	/	/

The original data were presented in Tables S8–S13.

## Data Availability

The assembled sequences can be found with CNGBdb accession numbers of CNA0146545, CNA0146546, CNA0146547, and CNA0146548.

## Supplementary Materials

The following supporting information can be downloaded at: https://www.mdpi.com/article/10.3390/toxins17040167/s1. Table S1: Insecticidal activity of Bt B14D2 strain against *M. hieroglyphica*; Table S2: Insecticidal activity of Bt B14D2 strain against *M. hieroglyphica*; Table S3: Insecticidal activity of Bt B14D2 strain against *Plutella xylostella*; Table S4: Bioassay of Vpb4Fa1 protein against *M. hieroglyphica*; Table S5: Bioassay of adult *M. hieroglyphica*; Table S6: LC_50_ bioassay of Vpb4Fa1 mutant protein against adult *M. hieroglyphica*; Table S7: Bioassay of larvae *M. hieroglyphica*; Table S8: Bioassay of adult *M. hieroglyphica*; Table S9: Bioassay of adult *M. hieroglyphica*; Table S10: Bioassay of adult *M. hieroglyphica*; Table S11: Bioassay of adult *M. hieroglyphica*; Table S12: Bioassay of adult *M. hieroglyphica*; Table S13: Bioassay of adult *M. hieroglyphica*; Figure S1: Supernatant of 75 Vpb4 Mutant Proteins by SDS-PAGE.
