# Supplementary material for: A Novel Vpb4 Gene and Its Mutants Exhibiting High Insecticidal Activity Against the Monolepta hieroglyphica
Source: Toxins (Basel). 2025 Apr 1;17(4):167. doi: 10.3390/toxins17040167 (PMC12031524; doi:10.3390/toxins17040167)
Supplement: Supplementary file 1 [file toxins-17-00167-s001.zip › toxins-3499815-Figure S1.pdf]

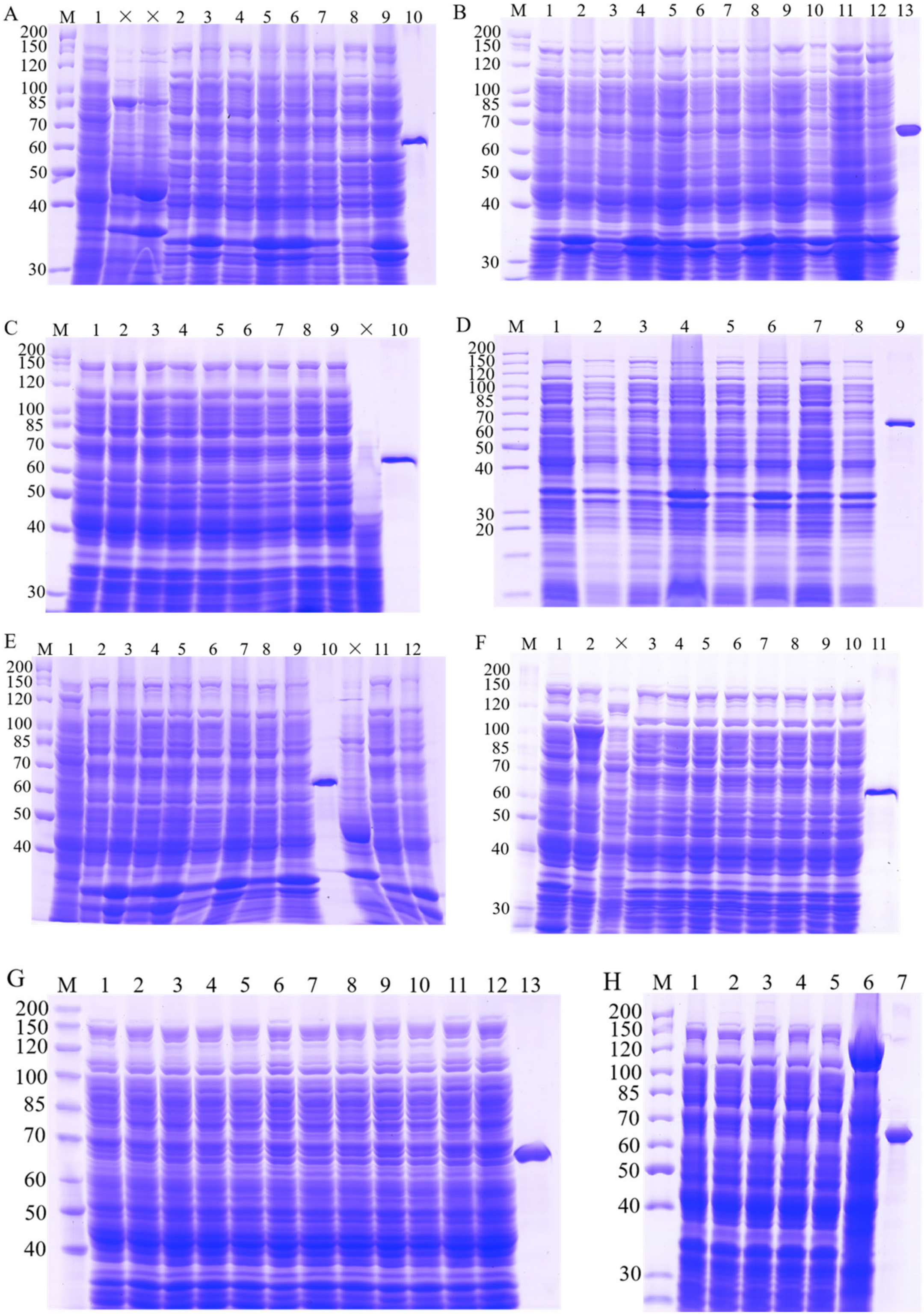

Figure S1 Supernatant of 75 Vpb4 Mutant Proteins by SDS-PAGE.

(A) M: Marker 26614; 1: pET-28a; 2: 2B8; 3: 3-41; 4: H4; 5: 2C2; 6: 4H10; 7: 4F7; 8: 4A6; 9: 2B12; 10: BSA; (B) M: 26614; 1: H4; 2: F3; 3: E6; 4: F6; 5: 2C10; 6: 6A2; 7: 4E12; 8: B7; 9: 5C8; 10: 4G10;11: 4H12; 12: 4C10; 13: BSA; (C) M: 26614; 1: 5E5; 2: 5G3; 3: 5E11; 4: 5F5; 5: 6B12; 6: G4; 7: 5B6; 8: 5A8; 9: 6A6; 10: BSA; (D) M: 26614; 1: 5E10; 2: 5E7; 3: 5A4; 4: 4F9; 5: 6B9; 6: 6A9; 7: 6D4; 8: 6C7; 9: BSA; (E) M: 26614; 1: pET-28a; 2: 5B10; 3: 5B6; 4: 6A7; 5: 4E6; 6: 5B4; 7: 6E6; 8: 6A11; 9:

6B7; 10: BSA; 11: 6E10; 12: 6C1; (F) M: 26614; 1: 6C9; 2: 5B7; 3: 5C9; 4: 6B5; 5: 6D11; 6: 4G7; 7: 6A8; 9: 6B1; 9: 6D6; 10: 6B6; 11: BSA;(G) M: 26614; 1: 6E9; 2: 6B4; 3: 4E4; 4: 6C5; 5: 6D7; 6: 4C6; 7: 6D8; 8: G3; 9: C5; 10: C9; 11: 3-2; 12: 4H6; 13: BSA; (H) M: 26614; 1: 6A5; 2: 6A3; 3: 6E4; 4: 4A5; 5: 6C2; 6: 6E2; 7: BSA
