# Supplementary material for: A Novel Vpb4 Gene and Its Mutants Exhibiting High Insecticidal Activity Against the Monolepta hieroglyphica
Source: Toxins (Basel). 2025 Apr 1;17(4):167. doi: 10.3390/toxins17040167 (PMC12031524; doi:10.3390/toxins17040167)
Supplement: Supplementary file 1 [file toxins-17-00167-s001.zip › toxins-3499815-supplementary.pdf]

Table S1: Insecticidal activity of Bt B14D2 strain against *M. hieroglyphica.*

Treatment	Total count			Survival count			Death count			Mortality (%)			Corrected mortality (%)		
	1	2	3	1	2	3	1	2	3	1	2	3	1	2	3
CK*	15	15	15	12	12	12	3	3	3	20	20	20	/	/	/
B14D2*	15	15	15	9	8	9	6	7	6	40	46.7	40	20	26.7	20
CK**	20	20	20	19	17	16	1	3	4	5	15	20	/	/	/
B14D2**	20	20	20	5	8	6	6	7	9	40	45	60	30.8	36.5	53.8

Note: * *M. hieroglyphica* adults; ** *M. hieroglyphica* larvae.

the concentration of Bt B14D2 strain at 10^8 cfu/mL.

CK: *M. hieroglyphica* adults were fed with sterilized water.

Table S2: Insecticidal activity of Bt B14D2 strain against *Mythimna separata and Helicoverpa armigera.*

Insect	Total count		Survival count		Death count		Mortality (%)		Corrected mortality (%)	
	1	2	1	2	1	2	1	2	1	2
CK	24	24	23	20	1	4	4.17	16.7	/	/
Mythimna separata	24	24	2	1	22	23	91.7	95.8	93.6	93.0
CK	20	20	17	20	3	0	15	0	/	/
Helicoverpa armigera	20	20	4	6	16	14	80	70	78.4	67.6

Note: The protein concentration is 10 μg/g.

CK: *Mythimna separata* were fed with 50 mmol/L Na_2CO_3 (pH 10.0).

Table S3: Insecticidal activity of Bt B14D2 strain against *Plutella xylostella.*

Treatment	Total count			Survival count			Death count			Mortality (%)			Corrected mortality (%)		
	1	2	3	1	2	3	1	2	3	1	2	3	1	2	3
CK	30	30	30	27	29	0	3	1	0	10.0	3.3	0	/	/	/
B14D2	30	30	30	0	0	1	30	30	29	100.0	100.0	98.9	100.0	100.0	96.5

Note: The protein concentration is 10 μg/g.

CK: *Mythimna separata* were fed with 50 mmol/L Na_2CO_3 (pH 10.0).

Table S4: Bioassay of Vpb4Fa1 protein against *M. hieroglyphica*

Treatment	Total count			Survival count			Death count			Mortality (%)			Corrected mortality (%)		
	1	2	3	1	2	3	1	2	3	1	2	3	1	2	3
CK*	15	15	15	12	12	13	3	3	2	20	20	13.3	/	/	/
Vp4Fa1*	15	15	15	8	7	6	7	8	9	46.7	53.3	60	35.3	42.8	51.4
CK**	15	15	15	12	12	12	3	3	3	20	20	20	/	/	/
Vpb4Fa1**	15	15	15	9	11	9	6	4	6	40	26.7	40	25	8.3	25

Note: * *M. hieroglyphica* adults; ** *M. hieroglyphica* larvae.

the concentration of Vpb4Fa1 protein is 40 μg/g.

CK: *M. hieroglyphica* adults were fed with 20 mM Tris-HCL.

Table S5: Bioassay of adult *M. hieroglyphica.*

Treatment	Corrected Mortality (Mean±SD, %)	Mutation site	The mutated amino acids	Domain
2B8	38.89±12.73	15	V-A	I
		212	T-A	
		311	S-P	II
		327	S-G	
		365	S-L	
3-41	30.56±4.81	304	E-X	II
		306	V-X	
		311	S-P	
		322	N-X	
		612	K-R	III
		726	T-X	IV
4H10	27.78±19.25	311	S-P	II
		416	I-M	
		600	Q-R	III
		741	N-S	IV
4F7	25±14.43	42	K-R	I
		198	N-S	
		311	S-P	II
		920	K-R	VI
4A6	5.56±4.81	227	T-A	I
		311	S-P	II
		743	N-D	IV
2B12	16.67±16.67	311	S-P	II
		346	F-S	
		617	L-P	III
H4	40.54±4.68	84	I-V	I
		311	S-P	II
F3	27.84±16.28	311	S-P	II
		790	V-A	V
E6	26.14±1.54	311	S-P	II
		415	S-P	
		444	I-T	
		614	G-D	III
		820	H-D	V

Continue

Treatment	Corrected Mortality (Mean±SD，%)	Mutation site	The mutated amino acids	Domain
F6	13.51±12.39	32	N-D	I
		63	I-T	
		250	S-P	
		311	S-P	II
		591	K-R	III
2C10	5.56±4.81	288	Y-C	I
		311	S-P	III
		674	Y-H	IV
6A2	16.67±8.33	311	S-P	II
		536	E-G	III
4E12	8.33±8.33	30	S-G	I
		180	E-G	
		311	S-P	II
B7	5.56±9.62	84	I-V	I
		127	E-G	
		226	Y-H	
		311	S-P	II
		925	N-T	VI
5C8	5.56±9.62	141	K-N	I
		200	F-S	
		311	S-P	II
		354	H-R	
4G10	8.33±14.43	185	L-P	I
		311	S-P	II
		552	I-V	III
		749	D-G	V
5E5	11.11±12.73	311	S-P	II
		507	T-A	
		787	Y-C	
5G3	19.44±17.35	311	S-P	II
		316	T-I	
		732	D-G	IV
5E11	22.22±12.73	311	S-P	II
		507	T-A	
		786	P-C	V
5F5	30.56±9.62	170	Q-L	I
		311	S-P	II
		618	T-A	III
		634	T-A	IV
		848	I-L	VI

Continue

Treatment	Corrected Mortality（Mean±SD，%）	Mutation site	The mutated amino acids	Domain
6B12	22.22±12.73	311	S-P	II
		495	V-I	
G4	30.56±17.35	109	K-E	I
		232	Q-R	
		311	S-P	II
5B6	8.33±14.43	71	L-R	I
		311	S-P	II
6A6	13.89±4.81	311	S-P	II
		945	I-F	VI
5E10	2.78±4.81	311	S-P	II
		651	R-G	IV
		958	K-R	VI
5E7	19.44±9.62	311	S-P	II
		901	Q-R	VI
5A4	25±14.43	149	D-G	I
		311	S-P	II
		507	T-A	
		538	D-N	III
		831	T-A	V
		894	K-N	VI
4F9	17.24±17.92	311	S-P	II
		558	A-T	III
6B9	41.38±11.95	311	S-P	II
		945	I-F	VI
6A9	55.17±15.8	311	S-P	II
		536	E-G	III
6D4	42.42±13.89	311	S-P	II
		399	Y-S	
		416	I-T	
		684	T-A	IV
		871	K-N	VI
6C7	30.41±5.16	311	S-P	II
		839	G-S	V
5B10	20.69±5.97	311	S-P	II
5B6	17.24±10.34	71	L-R	I
		311	S-P	II

Continue

Treatment	Corrected Mortality (Mean±SD，%)	Mutation site	The mutated amino acids	Domain
4E6	27.59±17.92	311	S-P	II
		564	T-A	III
		623	E-K	
		624	I-V	
		695	V-A	IV
		877	D-G	VI
5B4	11.49±9.95	311	S-P	II
		347	T-I	
6E6	55.17±5.97	311	S-P	II
		839	G-S	V
6A11	11.49±14.36	311	S-P	II
		399	Y-S	
		416	I-T	
		684	T-A	IV
		871	K-N	VI
6B7	34.48±15.8	311	S-P	II
		399	Y-S	
		436	Y-C	
		684	T-A	IV
		871	K-N	VI
6E10	31.03±5.97	112	I-T	I
		311	S-P	II
		374	I-V	
		402	R-C	
6C1	17.24±10.34	311	S-P	II
		481	N-T	
		744	I-V	IV
		842	N-D	V
6C9	2.3±3.98	311	S-P	II
		784	I-K	V
		865	W-R	VI
5B7	13.79±5.97	311	S-P	II
		347	T-I	
6E2	5.95±7.43	311	S-P	II
		387	N-D	
		911	S-G	VI
		965	G-C	
5C9	4.76±8.25	311	S-P	II
		316	T-I	
		732	D-G	IV

Continue

Treatment	Corrected Mortality （Mean±SD，%）	Mutation site	The mutated amino acids	Domain
6B5	50±16.37	311	S-P	
		399	Y-S	II
		416	I-T	
		684	T-A	IV
		871	K-N	VI
6D11	32.14±16.37	182	E-G	I
		311	S-P	II
		617	I-V	III
6A8	17.86±16.37	311	S-P	II
		362	I-T	
6B1	53.57±16.37	311	S-P	II
		569	N-D	III
6D6	14.39±10.71	311	S-P	II
		839	G-S	V
6B6	21.43±16.37	311	S-P	
		343	L-Q	II
		424	N-D	
		682	S-P	IV
6E9	10.71±6.19	311	S-P	II
		536	E-G	III
6B4	42.86±6.19	111	N-D	
		126	M-V	I
		233	I-T	
		311	S-P	II
		604	K-E	III
		878	V-I	VI
4E4	25±10.71	271	Y-C	I
		311	S-P	II
		620	H-R	III
		756	W-R	IV
6C5	39.29±12.37			I
		205	I-V	II
		311	S-P	VI
				II
6D8	21.43±12.37	311	S-P	II
		839	G-S	V
G3	57.14±10.71	311	S-P	II
		605	K-R	III

Continue

Treatment	Corrected Mortality（Mean±SD，%）	Mutation site	The mutated amino acids	Domain
C5	21.43±6.19	43	G-S	I
		300	F-I	II
		311	S-P	
		846	T-A	V
3-2	28.57±6.19	306	V-X	II
		311	S-P	
		353	G-E	
		491	T-X	
		496	T-X	
		500	K-X	
		537	N-D	III
		680	V-A	IV
		713	E-G	
		830	I-T	V
		878	V-A	VI
4H6	25±10.71	8	缺失	I
		311	S-P	II
		672	T-A	IV
		920	K-R	VI
6A5	21.43±6.19	271	Y-C	I
		311	S-P	II
		620	H-R	III
		756	W-R	IV
6A3	3.7±6.42	311	S-P	II
		517	T-A	III
		584	K-E	
		625	N-D	
		672	T-I	IV
		866	D-G	VI
		958	K-R	
6E4	11.11±11.11	182	E-G	I
		311	S-P	II
		617	I-V	III
4A5	48.15±6.42	97	C-H	I
		311	S-P	II
WT	27.78±11.71	/	/	/

Table S6: LC_{50} bioassay of Vpb4Fa1 mutant protein against adult *M. hieroglyphica.*

Treatment	Concentration (μg/g)	Total count			Survival count			Death count		
		1	2	3	1	2	3	1	2	3
C9	1	15	15	15	11	11	12	4	4	3
	2	15	15	15	11	11	10	4	4	5
	4	15	15	15	7	9	9	8	6	6
	8	15	15	15	6	8	7	9	7	8
	16	15	15	15	5	6	5	10	9	10
	32	15	15	15	2	1	4	13	14	11
6C2	1.75	15	15	15	11	9	11	4	6	4
	2.25	15	15	15	10	9	9	5	6	6
	4.5	15	15	15	7	8	7	8	7	8
	9	15	15	15	6	7	7	9	8	8
	18	15	15	15	4	5	5	11	10	10
	36	15	15	15	4	0	3	11	15	12
6A7	1.75	15	15	15	11	11	10	4	4	5
	2.25	15	15	15	10	10	7	5	5	8
	4.5	15	15	15	9	8	8	6	7	7
	9	15	15	15	7	8	6	8	7	9
	18	15	15	15	5	6	5	10	9	10
	36	15	15	15	0	4	1	15	11	14
CK		15	15	15	12	12	12	3	3	3

Note: CK: *M. hieroglyphica* adults were fed with 20 mM Tris-HCL.

Table S7: Bioassay of larvae *M. hieroglyphica.*

Treatment	Total count			Survival count			Death count			Mortality (%)			Corrected mortality (%)		
	1	2	3	1	2	3	1	2	3	1	2	3	1	2	3
C9	15	15	15	6	7	8	9	8	7	60	53.3	46.7	50	41.7	33.3
6C2	15	15	15	6	5	7	9	10	8	60	66.7	53.3	50	58.3	41.7
6A7	15	15	15	7	7	9	8	8	6	53.3	53.3	40	41.7	41.7	25
CK	15	15	15	12	12	12	3	3	3	20	20	20	/	/	/

Note: The protein concentration is 10 μg/g.

CK: *M. hieroglyphica* larvae were fed with 20 mM Tris-HCL.

Table S8: Bioassay of adult *M. hieroglyphica.*

Treatment	Total count			Survival count			Death count			Mortality (%)			Corrected mortality (%)		
	1	2	3	1	2	3	1	2	3	1	2	3	1	2	3
CK	15	15	15	13	12	11	2	3	4	13.3	20.0	26.7	/	/	/
2B8	15	15	15	7	6	9	8	9	6	53.3	60.0	40.0	41.7	50.0	25
3-41	15	15	15	8	9	8	7	6	7	46.7	40.0	46.7	33.3	25	33.3
H4	15	15	15	11	12	13	4	3	2	26.7	20	13.3	8.3	0	0
2C2	15	15	15	10	12	13	5	3	2	33.3	20	13.3	16.7	0	0
4H10	15	15	15	10	10	6	5	5	9	33.3	33.3	60	16.7	16.7	50
4F7	15	15	15	8	11	8	7	4	7	46.7	26.7	46.7	33.3	8.3	33.3
4A6	15	15	15	11	12	11	4	3	4	26.7	20	26.7	8.3	0	8.3
2B12	15	15	15	12	8	10	3	7	5	20	46.7	33.3	0	33.3	16.7
WT	15	15	15	10	11	9	5	4	6	33.3	26.7	40	16.7	8.3	25

Table S9: Bioassay of adult *M. hieroglyphica.*

Treatment	Total count			Survival count			Death count			Mortality (%)			Corrected mortality (%)		
	1	2	3	1	2	3	1	2	3	1	2	3	1	2	3
CK	15	15	15	12	12	13	3	3	2	20	20	13.3	/	/	/
H4	15	15	15	7	7	8	8	8	7	53.3	53.3	46.7	43.2	43.2	35.1
F3	15	15	15	8	7	11	7	8	4	46.7	53.3	26.7	29.5	43.2	10.8
E6	15	15	15	10	9	9	5	6	6	33.3	40	40	24.4	27.0	27.0
F6	15	15	15	9	11	12	6	4	3	40	26.6	20	27.0	10.8	2.7

Table S10: Bioassay of adult *M. hieroglyphica.*

Treatment	Total count			Survival count			Death count			Mortality (%)			Corrected mortality (%)		
	1	2	3	1	2	3	1	2	3	1	2	3	1	2	3
CK	15	15	15	13	11	12	2	4	3	13.3	26.7	20	/	/	/
2C10	15	15	15	13	11	11	2	4	4	13.3	26.7	26.7	0	8.3	8.3
6A2	15	15	15	11	9	10	4	6	5	26.7	40	33.3	8.3	25	16.7
4E12	15	15	15	13	11	10	2	4	5	13.3	26.7	33.3	0	8.3	16.7
B7	15	15	15	13	10	12	2	5	3	13.3	33.3	20	0	16.7	0
5C8	15	15	15	10	12	13	5	3	2	33.3	20	13.3	16.7	0	0
4G10	15	15	15	14	13	9	1	2	6	6.7	13.3	40	0	0	25
4H12	15	15	15	12	14	12	3	1	3	20	6.7	20	0	0	0
4C10	15	15	15	4	4	3	11	11	12	73.3	73.3	80	66.7	66..7	75
5E5	15	15	15	9	12	11	6	3	4	40	20	26.7	25	0	8.3
5G3	15	15	15	9	8	12	6	7	3	40	46.7	20	25	33.3	0
5E11	15	15	15	8	11	9	7	4	6	46.7	26.7	40	33.3	8.3	25
5F5	15	15	15	9	7	9	6	8	6	40	53.3	40	25	41.7	25
6B12	15	15	15	8	11	9	7	4	6	46.7	26.7	40	33.3	8.3	25
G4	15	15	15	9	6	10	6	9	5	40	60	33.3	25	50	16.7
5B6	15	15	15	12	9	12	3	6	3	20	40	20	0	25	0
5A8	15	15	15	13	12	12	2	3	3	13.3	20	20	0	0	0
6A6	15	15	15	10	11	10	5	4	5	33.3	26.7	33.3	16.7	8.3	16.7
5E10	15	15	15	11	12	12	4	3	3	26.7	20	20	8.3	0	0

continue

Treatment	Total count			Survival count			Death count			Mortality (%)			Corrected mortality (%)		
	1	2	3	1	2	3	1	2	3	1	2	3	1	2	3
5A4	15	15	15	8	11	8	7	4	7	46.7	26.7	46.7	33.3	8.3	33.3
5E7	15	15	15	9	9	11	6	6	4	40	40	26.7	25	25	8.3

Table S11: Bioassay of adult *M. hieroglyphica.*

Treatment	Total count			Survival count			Death count			Mortality (%)			Corrected mortality (%)		
	1	2	3	1	2	3	1	2	3	1	2	3	1	2	3
CK	11	11	11	11	10	8	0	1	3	0	9.1	27.3	/	/	/
4F9	11	11	11	9	9	6	2	2	5	18.2	18.2	45.5	6.9	6.9	37.9
6B9	11	11	11	9	11	9	6	4	6	54.5	36.4	54.5	48.3	27.6	48.3
6A9	11	11	11	6	3	4	5	8	7	45.5	72.7	63.6	37.9	67.0	58.6
6D4	11	11	11	5	6	8	6	5	3	54.5	45.5	27.3	48.3	37.9	17.2
6C7	11	11	11	8	7	7	3	4	4	27.3	36.4	36.4	17.2	27.6	27.6
6C2	11	11	11	2	4	5	9	7	6	81.8	63.6	54.5	79.3	58.6	48.3
5B10	11	11	11	7	8	8	4	3	3	36.4	27.3	27.3	27.6	17.2	17.2
5B6	11	11	11	8	9	7	3	2	4	27.3	18.2	36.4	17.2	6.9	27.6
6A7	11	11	11	2	4	4	9	7	7	81.8	63.4	63.4	79.3	58.6	58.6
4E6	11	11	11	5	8	8	6	3	3	54.5	27.3	27.3	48.3	17.2	17.2
5B4	11	11	11	10	8	8	1	3	3	9.1	27.3	27.3	0	17.2	17.2
6E6	11	11	11	4	5	4	7	6	7	63.6	54.5	63.6	58.6	48.3	58.6
6A11	11	11	11	10	7	9	1	4	2	9.1	36.4	18.2	0	27.6	6.9
6B7	11	11	11	5	6	8	6	5	3	54.5	45.5	27.3	48.3	37.9	17.2
6E10	11	11	11	7	7	6	4	4	5	36.4	36.4	45.5	27.6	27.6	37.9
6C1	11	11	11	9	7	8	2	4	3	18.2	36.4	27.3	6.9	27.6	17.2
6C9	11	11	11	9	10	10	2	1	1	18.2	9.1	9.1	6.9	0	0
5B7	11	11	11	8	9	8	3	2	3	27.3	18.2	17.3	17.2	6.9	17.2

Table S12: Bioassay of adult *M. hieroglyphica.*

Treatment	Total count			Survival count			Death count			Mortality (%)			Corrected mortality (%)		
	1	2	3	1	2	3	1	2	3	1	2	3	1	2	3
CK	11	11	11	9	9	10	2	2	1	18.2	18.2	9.1	/	/	/
6E2	11	11	11	10	9	8	1	2	3	9.1	18.2	27.3	0	3.6	14.3
5C9	11	11	11	8	10	10	3	1	1	27.3	9.1	9.1	14.3	0	0
6B5	11	11	11	3	6	5	8	5	6	72.7	45.5	54.5	67.9	35.7	46.4
6D11	11	11	11	8	5	6	3	6	5	27.3	54.5	45.5	14.3	46.4	35.7
4G7	11	11	11	3	4	4	8	7	7	72.7	63.6	63.6	67.9	57.1	57.1
6A8	11	11	11	9	8	6	2	3	5	18.2	27.3	45.5	3.6	14.3	35.7
6B1	11	11	11	4	3	6	7	8	5	63.6	72.7	45.5	57.1	67.9	35.7
6D6	11	11	11	8	7	9	3	4	2	27.3	36.4	18.2	14.3	25	3.57
6B6	11	11	11	9	7	6	2	4	5	18.2	36.4	45.5	3.6	25	35.7
6E9	11	11	11	9	8	8	2	3	3	18.2	27.3	27.3	3.66	14.3	14.3
6B4	11	11	11	5	5	6	6	6	5	54.5	54.5	45.5	46.4	46.4	35.7
4E4	11	11	11	7	8	6	4	3	5	36.4	27.3	45.5	25	14.3	35.7

continue

Treatment	Total count			Survival count			Death count			Mortality (%)			Corrected mortality (%)		
	1	2	3	1	2	3	1	2	3	1	2	3	1	2	3
6D7	11	11	11	2	3	5	9	8	6	81.8	72.7	54.5	78.6	67.9	46.4
4C6	11	11	11	5	3	3	6	8	8	54.5	72.7	72.7	46.4	67.9	67.9
6D6	11	11	11	8	8	6	3	3	5	27.3	27.3	45.5	14.3	14.3	35.7
G3	11	11	11	4	5	3	7	6	8	63.6	54.5	72.7	57.1	46.4	67.9
C5	11	11	11	7	8	7	4	3	4	36.4	27.3	36.4	25	14.3	25
C9	11	11	11	1	3	1	10	8	10	90.9	72.7	90.9	89.3	67.9	89.3
3-2	11	11	11	6	7	7	5	4	4	45.5	36.4	36.4	35.7	25	25
4H6	11	11	11	7	6	8	4	5	3	36.4	45.5	27.3	25	35.7	14.3
6A5	11	11	11	8	7	7	3	4	4	27.3	36.4	36.4	14.3	25	25
6C5	11	11	11	7	5	5	4	6	6	36.4	54.5	54.5	25	46.4	46.4

Table S13: Bioassay of adult *M. hieroglyphica.*

Treatment	Total count			Survival count			Death count			Mortality (%)			Corrected mortality (%)		
	1	2	3	1	2	3	1	2	3	1	2	3	1	2	3
CK	11	11	11	9	9	9	2	2	2	18.2	18.2	18.2	/	/	/
6A3	11	11	11	8	9	9	3	2	2	27.3	18.2	18.2	11.1	0	0
6E4	11	11	11	8	9	7	3	2	4	27.3	18.2	36.4	11.1	0	22.2
4A5	11	11	11	5	5	4	6	6	7	54.5	54.5	63.6	44.4	44.4	55.6

Note: The concentration of Vpb4Fa1 protein is 10 μg/g.

CK: *M. hieroglyphica* adults were fed with 20 mM Tris-HCL.
